# Supplementary figures and images for: Mini-XT, a miniaturized tagmentation-based protocol for efficient sequencing of SARS-CoV-2
Source: J Transl Med. 2022 Mar 3;20:105. doi: 10.1186/s12967-022-03307-9 (PMC8892412; doi:10.1186/s12967-022-03307-9)

Figure S1

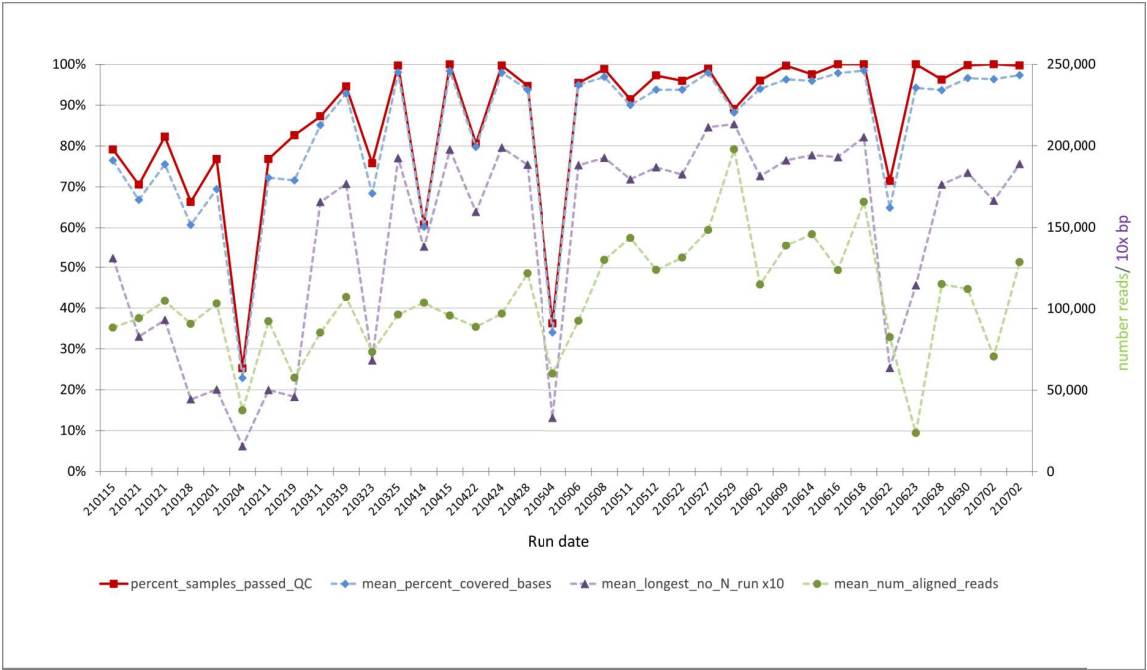

Figure S2

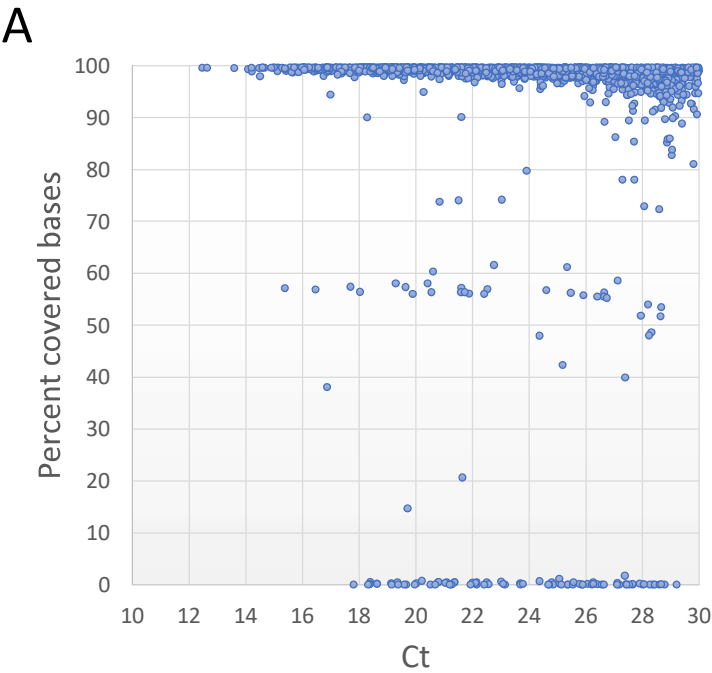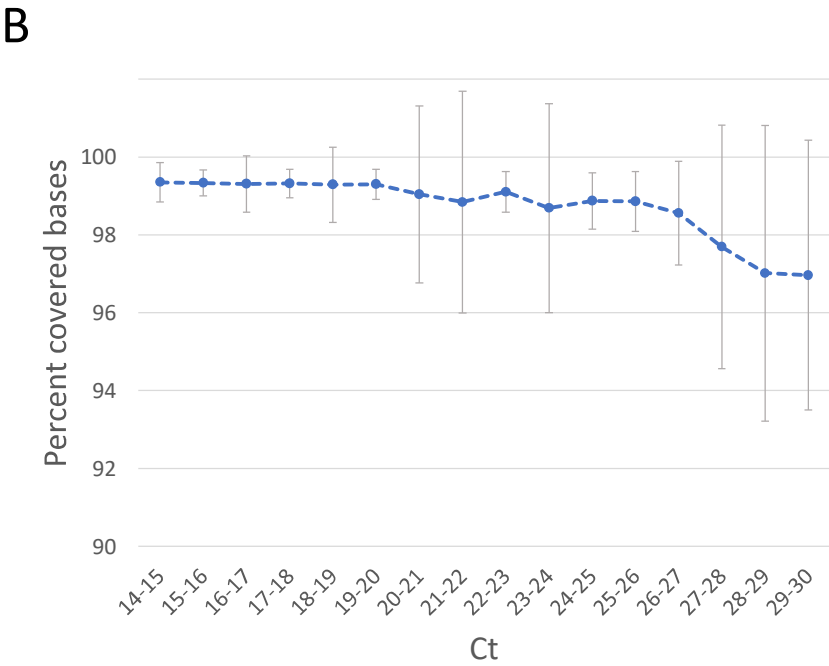

Figure S3

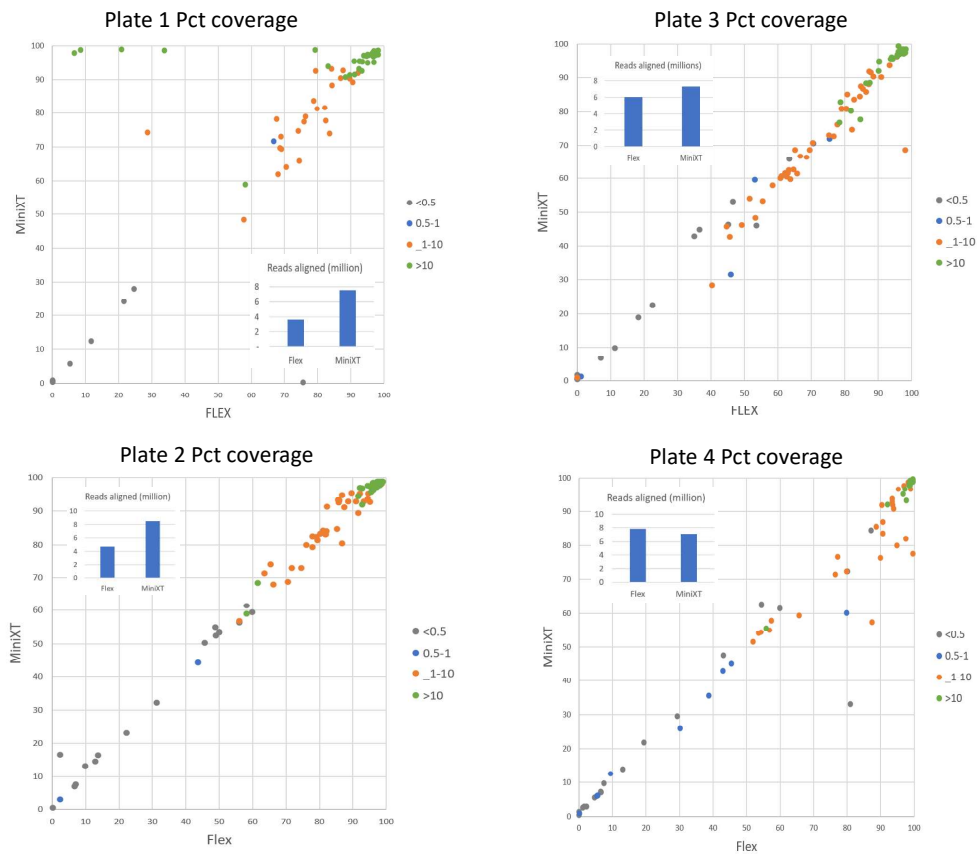

Figure S4

B.1.177.5 & B.1.177.86

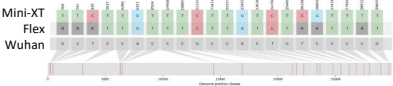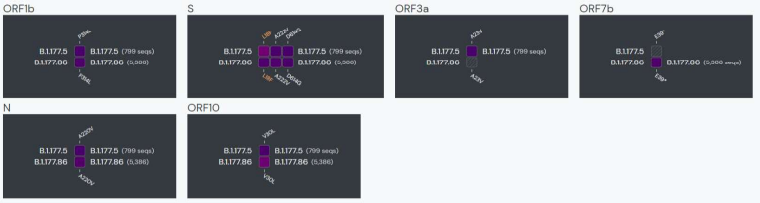

B.1.177.54 & B.1.177.55

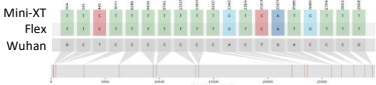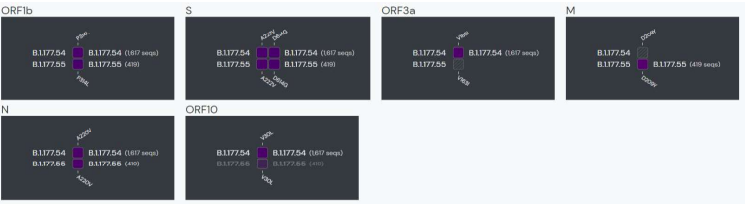

B.1.1 & B.1.36.36

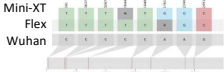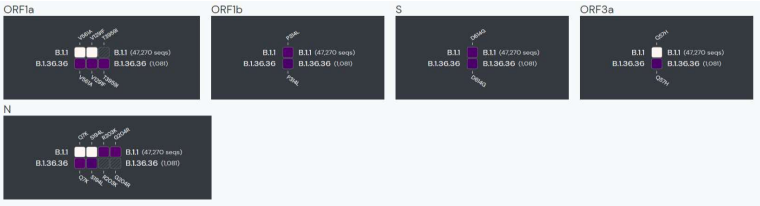

V.2 (B.1.177.54.2) & B.1.177.24

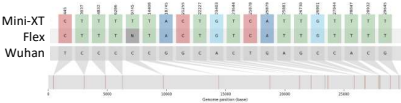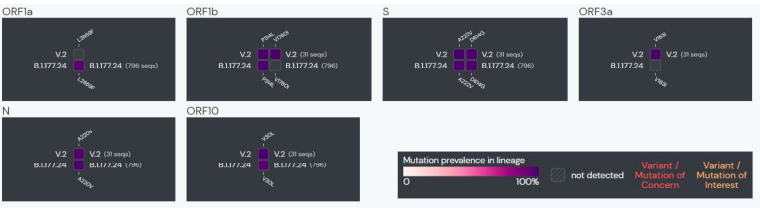

Figure S5

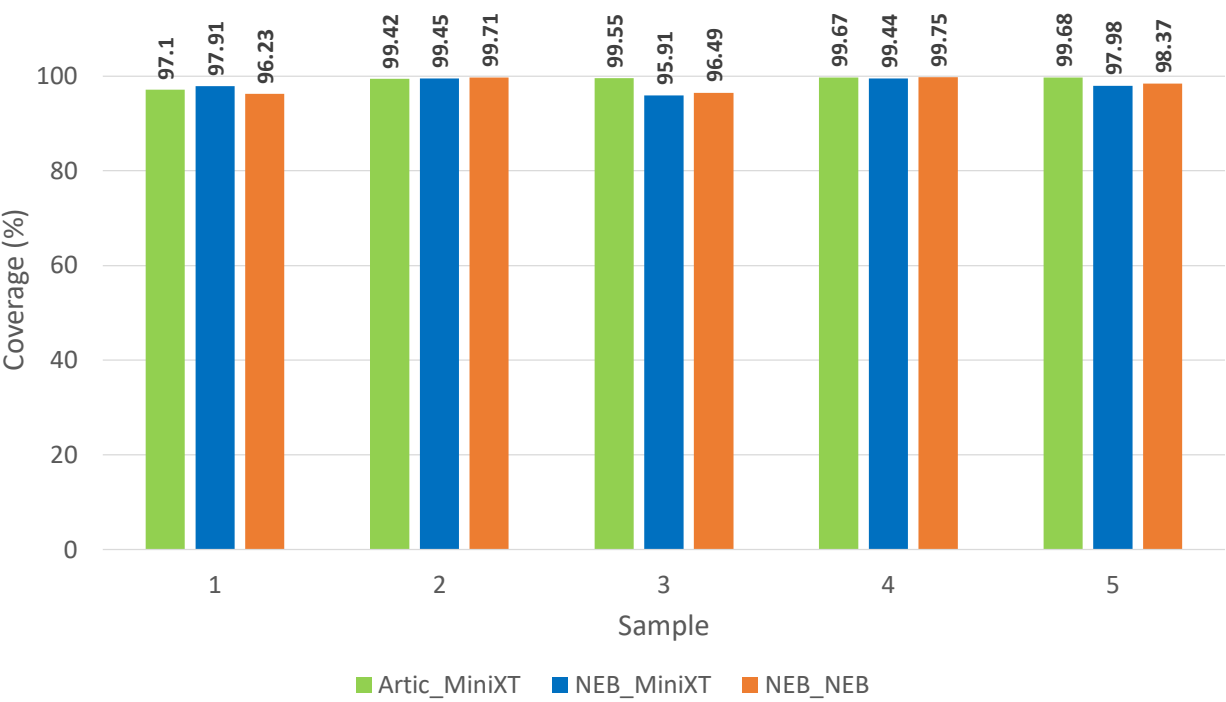

Supplement: Supplementary file 2 — Additional file 2: Figure S1. Mini-XT sequencing metrics for all individual Mini-XT runs indicated by date (YYMMDD). Figure S2. RT-qPCR Ct values of the SARS-CoV-2 positive RNA samples sequenced using Mini-XT vs. percent of bases covered. A Relationship between Ct value (Orf1ab target gene) and the percent of bases covered in SARS-CoV-2 genome sequences for 2000 samples processed with the Mini-XT protocol. B Average percent covered bases (± SD) for samples with increasing Ct ranges (excluding samples with one or both amplicon pools failing, ie percent covered bases < 70). Figure S3. Comparison of individual 96 well plates of samples prepared using the DNA Flex library kit with the same samples prepared with the Mini-XT protocol. The concentrations of the amplicon pools are indicated in ng/µl and broadly correlate with the percentage coverage achieved. The insets indicate the total number of aligned reads for each set of samples. Figure S4. Characteristic variants of sublineages called differentially between sequencing protocols. SNP-IT plots indicate that all the SNPs called from Mini-XT and Flex sequences are either shared or not called in one sequence. The comparison illustrates that most characteristic variants of the discrepant lineages are shared and only vary at several positions. Figure S5. Comparison of Arctic v3 and NEB primer pools. Samples are named according to the amplification and then library preparation method and ordered according to percentage coverage achieved with Articv3 primers and mini-XT library preparation (Artic_MiniXT). [file 12967_2022_3307_MOESM2_ESM.pdf]
